# Prohibited Bliss

**Published:** 1886-08

**Authors:** 


**Prohibited Bliss.**—While on his recent visit to Detroit Lake, William Penn Nixon, accompanied by his wife, visited White Earth Indian agency. One morning he was chatting with agent Sheehan, who was dilating upon the rapid progress made by the aborigines, when an Indian girl dashed up to the door upon a pretty pony, dismounted, and taking the train of riding habit on her arm, walked into the agency, presented a certificate from a surveyor in regard to fees paid and said she wished a patent for her land. "Here," said Agent Sheehan, "is a good illustration of just what we were speaking about." The Indian girl, though a full-blood, made an attractive picture in her bright riding habit, and the editor spoke up saying: "By the way, how can I secure some land here ?" "Where do you live?" asked the red girl. "In Chicago," replied the philosopher of the daily press. "Oh, then, you'll have to marry me in order to obtain land here on the reservation," piquantly spoke up the maid. "But I am already married ; here is my wife." "Yes, but she is old ; I am young. I hear you can easily get a divorce in Chicago. Come here ; marry me, and you can have land." Her entreaties were so earnest and arguments so strong that the editor was compelled to use his utmost strategy in order to prevent completing the "treaty" with the Indian maid.—*St. Paul Globe.*

**A student** in Michigan university wrote to a Kansas druggist about taking a place in his store as prescription clerk. This is what the druggist wrote back: "DEAR SIR yours Recd in Reply i will Give you a Brief Description of our Business Perhaps you understand the nature of a Drug Store in kansas we do some liquor business in a Back Room By the Drink our Prescription trade Runs from two to three thousand Pr year Some Clerks objects to the Back Room trade I give you the facts in the case so that you will not be Disappointed your Bord By the week will cost you from $3.50 to $5.00 a week now if you except this position answer by telegraph at once as I kneed a clark very Bad & must have one as Soon as Possible."

